# Thrombomodulin is associated with increased mortality and organ failure in mechanically ventilated children with acute respiratory failure: biomarker analysis from a multicenter randomized controlled trial

**DOI:** 10.1186/s13054-021-03626-1

**Published:** 2021-08-03

**Authors:** Ana Carolina Costa Monteiro, Heidi Flori, Mary K. Dahmer, Myung Shin Sim, Michael W. Quasney, Martha A. Q. Curley, Michael A. Matthay, Anil Sapru, for the BALI Study Investigators of the Pediatric Acute Lung Injury and Sepsis Investigators (PALISI) Network, Ana Carolina Costa Monteiro, Heidi Flori, Mary K. Dahmer, Myung Shin Sim, Michael W. Quasney, Martha A. Q. Curley, Michael A. Matthay, Anil Sapru, Scot T. Bateman, M. D. Berg, Santiago Borasino, G. Kris Bysani, Allison S. Cowl, Cindy Darnell Bowens, E. Vincent S. Faustino, Lori D. Fineman, A. J. Godshall, Ellie Hirshberg, Aileen L. Kirby, Gwenn E. McLaughlin, Shivanand Medar, Phineas P. Oren, James B. Schneider, Adam J. Schwarz, Thomas P. Shanley, Lauren R. Sorce, Edward J. Truemper, Michele A. Vander Heyden, Kim Wittmayer, Athena Zuppa, David Wypij

**Affiliations:** 1 Division of Pulmonary and Critical Care Medicine, Department of Medicine, UCLA Ronald Reagan Hospital, University of California, Los Angeles, 757 Westwood Plaza, Los Angeles, CA 90095 USA; 2 Division of Pediatric Critical Care Medicine, Department of Pediatrics and Communicable Diseases, University of Michigan, Ann Arbor, MI USA; 3 Division of General Internal Medicine and Health Services Research, University of California, Los Angeles, Los Angeles, CA USA; 4 Division of Anesthesia and Critical Care Medicine (Perelman School of Medicine), Department of Family and Community Health (School of Nursing), University of Pennsylvania, Philadelphia, PA USA; 5 Departments of Medicine and Anesthesia, Cardiovascular Research Institute, University of California, San Francisco, San Francisco, CA USA; 6 Division of Pediatric Critical Care, Department of Pediatrics, University of California, Los Angeles, Los Angeles, CA USA; 7 PALISI Network, Livingston, NJ USA; 8 University of Massachusetts Memorial Children’s Medical Center, Worcester, MA USA; 9 University of Arizona Medical Center, Tucson, AZ USA; 10 Children’s Hospital of Alabama, Birmingham, AL USA; 11 Medical City Children’s Hospital, Dallas, TX USA; 12 Connecticut Children’s Medical Center, Hartford, CT USA; 13 Children’s Medical Center of Dallas, Dallas, TX USA; 14 Yale-New Haven Children’s Hospital, New Haven, CT USA; 15 University of California San Francisco Benioff Children’s Hospital at San Francisco, San Francisco, CA USA; 16 Florida Hospital for Children, Orlando, FL USA; 17 Primary Children’s Medical Center, Salt Lake City, UT USA; 18 Oregon Health and Science University Doernbecher Children’s Hospital, Portland, OR USA; 19 Holtz Children’s Hospital, Jackson Health System, Miami, FL USA; 20 Cohen Children’s Medical Center of New York, Hyde Park, NY USA; 21 St. Louis Children’s Hospital, St. Louis, MO USA; 22 Children’s Hospital of Orange County, Orange, CA USA; 23 C. S. Mott Children’s Hospital at the University of Michigan, Ann Arbor, MI USA; 24 Ann & Robert H. Lurie, Children’s Hospital of Chicago, Chicago, IL USA; 25 Children’s Hospital and Medical Center, Omaha, NE USA; 26 Children’s Hospital at Dartmouth, Dartmouth, NH USA; 27 Advocate Hope Children’s Hospital, Park Ridge, IL USA; 28 Children’s Hospital of Philadelphia, Philadelphia, PA USA; 29 Department of Biostatistics, Harvard School of Public Health, Boston, MA USA; 30 Department of Pediatrics, Harvard Medical School, Boston, MA USA; 31 Department of Cardiology, Boston Children’s Hospital, Boston, MA USA

**Keywords:** Acute respiratory distress syndrome, Acute respiratory failure, Dead space ventilation, Thrombomodulin, Coagulation, Vascular injury

## Abstract

**Background:**

Acute respiratory failure (ARF) can progress to acute respiratory distress syndrome and death. Biomarkers may allow for risk stratification and prognostic enrichment in ARF. Thrombomodulin (TM) is a transmembrane antithrombotic mediator expressed in endothelial cells. It is cleaved into its soluble form (sTM) during inflammation and vascular injury. Levels of sTM correlate with inflammation and end organ dysfunction.

**Methods:**

This was a prospective observational study of 432 patients aged 2 weeks—17 years requiring invasive mechanical ventilation. It was ancillary to the multicenter clinical trial, Randomized Evaluation of Sedation Titration for Respiratory Failure (RESTORE). After consent, patients had up to 3 plasma samples collected at 24-h intervals within 5 days after intubation. sTM was assayed by ELISA. The Hazard ratio (HR) for 90-day mortality was determined by Cox regression. Mixed effect models (MEM) were used to test for association with extrapulmonary multiorgan failure (MOF) and oxygenation index (OI). Age, race, sex and PRISM-III scores were used as confounding variables for multivariable analyses.

**Results:**

sTM values ranged from 16.6 to 670.9 ng/ml within 5 days after intubation. Higher sTM was associated with increased 90-day mortality (*n* = 432, adjusted HR = 1.003, *p* = 0.02) and worse OI in the first 5 days after intubation (*n* = 252, Estimate = 0.02, *p* < 0.01). Both initial and slope of sTM were associated with increased extrapulmonary MOF in unadjusted and adjusted analyses (Intercept, Estimate = 0.003, *p* < 0.0001; and slope, Estimate = 0.01, *p* = 0.0009, *n* = 386).

**Conclusions:**

Plasma sTM is associated with mortality, severity of hypoxic respiratory failure and worsening extrapulmonary MOF in children with ARF. This suggests a role of vascular injury in the pathogenesis of ARF and provides potential applicability towards targeted therapies.

*Trial registration*: https://clinicaltrials.gov/ct2/show/NCT00814099.

In healthy lung endothelium, thrombomodulin (TM) recruits thrombin to activate Protein-C (PC/APC), that inhibits plasminogen activator-1 (PAI-1) and thrombosis. In inflamed and damaged endothelium, TM is cleaved into its soluble form (sTM), precluding its usual regulation of thrombosis. In this study, we measured plasma sTM levels in pediatric patients with respiratory failure and found that sTM correlated with mortality and other clinical markers of poor outcomes.
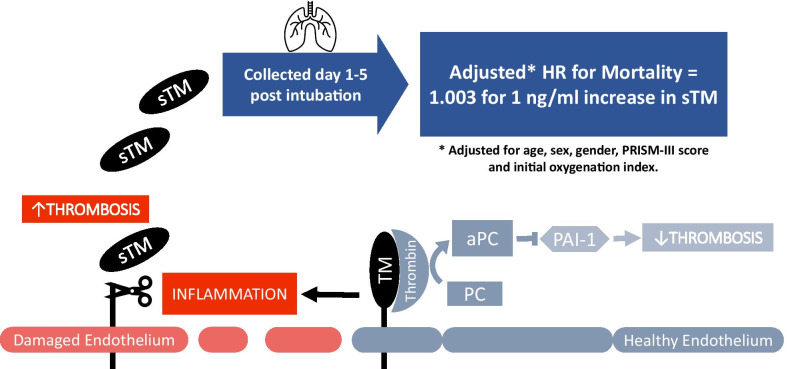

**Supplementary Information:**

The online version contains supplementary material available at 10.1186/s13054-021-03626-1.

## Background

Acute respiratory failure (ARF) can progress to pediatric acute respiratory distress syndrome (PARDS), multiorgan failure (MOF) and death [1–3]. The heterogeneity of ARF and PARDS are potential impediments to the discovery of effective therapeutic options [4], and consequently, recent studies have aimed to endotype, subclassify and prognostically enrich ARDS based on clinical and serum biomarkers [5, 6]. In adults, clinical markers such as dead space fraction [7–9] and the ventilatory ratio [10–12] have highlighted the contribution of inefficient ventilation in the prognosis of ARDS and are starting to be used in clinical investigation. Serum biomarkers, by enabling mechanism-specific subclassification of ARDS, may also elucidate pathway-targeted therapies and enable predictive enrichment [13]. A role for inflammation in the pathogenesis of PARDS has been supported by studies that showed plasma levels of interleukins (IL)-6, IL-8, IL-10, IL-18, soluble Tumor Necrosis Factor Receptor-2 and interleukin-1 receptor antagonist [14–16] are associated with higher mortality in these patients. In addition, plasminogen activator inhibitor-1, soluble thrombomodulin (sTM) and von Willebrand factor-antigen, involved in endothelial injury and dysregulated coagulation, are also implicated in the pathogenesis of adult [17–19] and pediatric [20, 21] ARDS, potentially through microvascular thrombosis contributing to dead space ventilation and organ dysfunction.

Soluble thrombomodulin is an attractive candidate biological marker for respiratory failure and ARDS because thrombomodulin, an anti-thrombotic agent found in the endothelial cell surface, is cleaved into its soluble form in response to local endothelial damage [22]. Both full length and the soluble form of thrombomodulin are protective against thrombosis; however, once thrombomodulin is cleaved and released into the circulation, it is assumed that the local anti-thrombotic effect is lost due to reduced avidity of the marker after cleavage, the generation of fragments of varying lengths and affinities, and whole-body redistribution. While it is likely that sTM levels increase in response to endothelial damage in a variety of organs, thrombomodulin is most prominently expressed in the human lung [22]. Thrombomodulin also plays an important role in lung development [23], which may imply a higher concentration of sTM in the pediatric lung, but this is not known.

Elevated levels of plasma sTM reflect inflammation, endothelial damage and loss of protection against thrombosis. A post hoc analysis of the FACTT trial revealed that elevated levels of plasma sTM were associated with higher mortality in adult patients with ARDS [19], and another study reported that specific gene polymorphisms of thrombomodulin have been associated with increased mortality in adult ARDS [24]. In children, a study in septic meningitis demonstrated the loss of local endothelial thrombomodulin and an elevation of plasma sTM [25]. In addition, we reported preliminary findings that sTM levels are associated with increased mortality in children with ARDS caused by indirect lung injury [21], though these findings have not yet been validated in an independent, heterogeneous cohort. A recent systematic review has highlighted the insufficient number of studies evaluating the role of sTM as a predictor of mortality in ARDS [26]. Therefore, as part of the *Genetic Variation and Biomarkers in Children with Acute Lung Injury* (*BALI*; R01HL095410) which enrolled over 500 patients who were part of the *Randomized Evaluation of Sedation Titration for Respiratory Failure* (*RESTORE;* U01 HL086622) prospective clinical trial, we tested the hypothesis that plasma sTM is a predictor of ARDS severity, mortality and worse outcomes in pediatric patients with acute respiratory failure requiring mechanical ventilation.

## Methods

### Patients

This study, *Genetic Variation and Biomarkers in Children with Acute Lung Injury* (*BALI*; R01HL095410), was an ancillary study to the multisite clinical trial, *Randomized Evaluation of Sedation Titration for Respiratory Failure* (*RESTORE*; U01 HL086622) that enrolled intubated mechanically ventilated children [16]. Details of the study methodology have been published previously [27], and relevant details are summarized in the appendix.

### Measurements

Blood samples were taken within 24 h of consent and again 24 and 48 h later, with the first blood sample drawn within three days of intubation (days 0–3) in most patients (98%). Plasma thrombomodulin levels were measured using two-antibody sandwich enzyme linked immunosorbent assays (ELISA, Asserchrome, Diagnostica Stago). The measurements were carried out in duplicate and followed the manufacturer’s protocol. For this study, we analyzed up to three sTM measurements per patient, collected within the first 5 days after intubation.

### Primary outcomes

We examined the association between plasma sTM and 90-day in-hospital mortality adjusted for confounding variables.

### Secondary outcomes

We examined the association of sTM with OI, the presence of non-pulmonary organ failure, ventilation-free days and PICU length of stay in survivors. We used the PALICC definition of ARDS. The determination of other secondary outcomes is as described in the supplement.

### Confounding variables

The main analysis was adjusted for age, race, sex and PRISM-III scores by multivariable analyses. Additional multivariable models incorporated OI from the first 24 h after intubation, use of vasopressors at day 1 and use of neuromuscular blockade at day 1. These confounders were chosen a priori for their clinical significance and face validity. We used PRISM-III to adjust for baseline severity of illness.

### Statistics

Given the unique nature of our dataset, which included repeated measurements of sTM along several days, and both continuous and binary outcomes with time varying covariates, we tested the relationship between sTM and primary and secondary outcomes using multiple approaches. We calculated odds ratio (OR) of mortality (alive or deceased at 90 days) given daily sTM level for days 0–2 by use of logistic regression. Receiver operating characteristic (ROC) curves were then evaluated to assess whether sTM drawn on these days could predict mortality. We also analyzed the relationship of sTM with mortality utilizing a composite estimate of all sTM levels in an individual patient using sTM intercept and slope. sTM intercept and sTM slope were determined by establishing a least square (LS) estimate between daily sTM values measured in the first 5 days. The intercept was the projected value of sTM where the LS line crossed t = 0. The slope indicates the rate of change of sTM in the first 5 days.

Finally, the hazard ratio (HR) for 90 day in-hospital mortality was assessed from sTM of all patient plasma samples collected between the day of intubation (day 0) and day 5 using counting process Cox proportional hazard model [28].

sTM values on individual days up to day 3 were compared by Mann–Whitney U test between patients with PARDS and those without (days 4 and 5 were excluded due to low numbers).

Mixed effect modelling (MEM) was used to test the relationship of sTM with MOF and PICU length of stay. MEM was also used to evaluate the relationship between the initial sTM (intercept) or the rate of increase in sTM (slope) and maximum OI. Daily OIs (or if unavailable, converted OSIs) up until maximum value were analyzed using mixed effect modelling (MEM), with sTM intercepts and slopes as predictor variables and age, gender, race/ethnicity and PRISM-III score as confounding variables. The relationship between initial sTM or rate of change of sTM and daily number of failed organs within the first 28 days was also evaluated using MEM.

The outcome of ventilation free days was analyzed using Fine and Gray model with Cox proportional hazard regression. Death was utilized as a competing risk.

### Study approval

Written informed consent was obtained from patients or their guardians prior to inclusion in the study. The study was approved by the Institutional Review Boards at all participating sites.

## Results

### Study population

In total, 549 patients were enrolled in the *BALI* study with 480 having plasma samples. Of those, 432 had one to three samples assayed for sTM within 5 days of intubation (day 0) (Additional file 2: Figure S1). These patients formed the population for this study. Clinical characteristics of the entire *BALI* cohort as well as those with and without PARDS have been described previously [15]. Clinical characteristics of the population for this study is shown in Additional file 1: Table S1. Mortality in the *BALI* cohort was 9%, with a median duration of mechanical ventilation of 7.1 days (IQR, 4.0–13.6) and a median PICU length of stay in survivors of 10.6 days (IQR, 6.6–18.4) [16]. The main primary cause of death was respiratory failure (17 patients, 4%), followed by multi organ failure (10 patients, 2.3%), as listed in Additional file 1: Table S2.

### Plasma soluble thrombomodulin increases with time

**Fig. 1:**
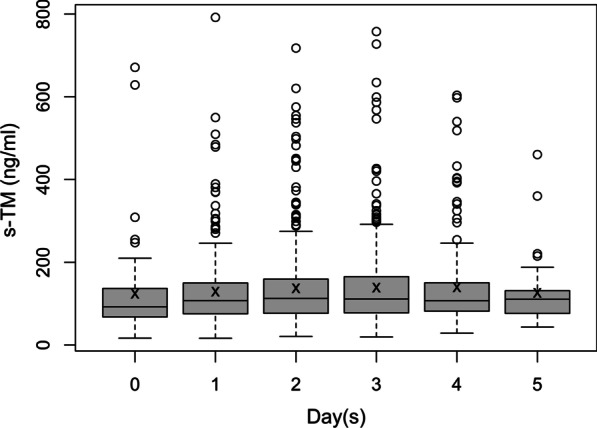
Box plot representing distribution of daily sTM values collected for each patient. Mean for each day is represented by ‘*x*’, outliers are represented by an open circle. Number (*n*) for individual days is as follows: 56 on day 0, 134 on day 1, 167 on day 2, 118 on day 3, 45 on day 4, 4 on day 5

One to three measurements of daily sTM were obtained within the first 5 days of the study for 432 patients (Additional file 2: Figure S1). Linear regression revealed that the rate of increase in sTM over the first 5 days was statistically significant, with an average daily increase of 5.00 ng/ml (*p* < 0.01). The distribution and the median values of sTM by day are illustrated in Fig. 1. The distribution of sTM on individual days was not statistically different between patients with or without PARDS (unadjusted, Additional file 2: Figure S2). The wide standard deviation for sTM was partly attributed to the inherent heterogeneity of the study population. As such, multivariate analyses were utilized to adjust for the effects of age, race and severity of illness.

### Soluble thrombomodulin correlates with increased mortality in ventilated pediatric patients

We performed univariate analysis using sTM as the predictor variable with mortality as the outcome, and multivariable analysis incorporating age, PRISM-III score, race (caucasian vs. not) and sex as covariates. Univariate, logistic regression analysis on individual days revealed that sTM measured at days 1 and 2 were associated with higher OR for mortality (day 1, OR = 1.005 per unit increase in sTM, CI = 1.001–1.008, *n* = 233 and day 2, OR = 1.004 per unit increase in sTM, CI = 1.002–1.007, *n* = 321, data not shown). Multivariable analysis of individual days revealed that sTM levels adjusted for selected covariates and measured at days 1 and 2 were associated with higher OR for mortality (1.01, *p* = 0.02 for day 1, Table 1, and *p* < 0.01 for day 2, data not shown).

**Table 1:** Soluble Thrombomodulin from Day 1 predicts Mortality

Covariates	OR (95% CI)	*P* value
sTM day 1	1.01 (1.00 – 1.01)	0.02
Age (Years)	1.12 (1.02 – 1.22)	0.02
Sex (Male)	0.68 (0.25 – 1.89)	0.46
Race (White v not)	0.89 (0.27 – 3.02)	0.86
PRISM-III Score	1.04 (0.98 – 1.12)	0.25

Multivariable logistic regression analysis of the Odds Ratio of Mortality based on selected covariates. sTM from day 1 was the selected predictor variable. *n* = 233

**Fig. 2:**
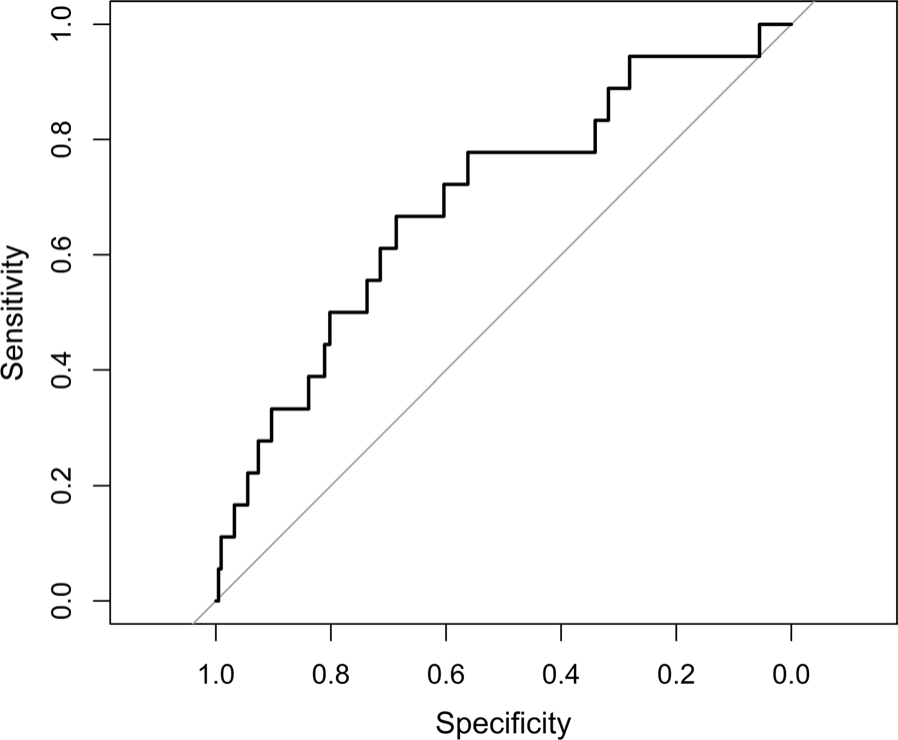
Receiver operating curve for the odds ratio of mortality based on sTM measured at day 1. *n* = 233

A receiver-operating characteristic (ROC) curve for the univariate analysis of sTM and mortality revealed an area under the curve (AUC) of 0.70 for thrombomodulin at day 1 (Fig. 2) and an AUC of 0.63 for day 2 (data not shown). Given the higher AUC, empirically selected values of day 1 sTM were assessed for their utility in predicting mortality. At day 1, we found that the level of sTM that would provide the optimal sensitivity and specificity based on ROC would be 130 ng/ml, which in this cohort provided a specificity of 69% and a sensitivity of 67% for its association with mortality. Additionally, at day 1, a cut off sTM level of 185 ng/ml would provide a specificity of 90%, however, a sensitivity of only 33% for correlation with mortality in this population, as assessed by ROC (Fig. 2). Conversely, a sTM cut-off value of 80 ng/ml would confer a sensitivity of 89% and a specificity of 31%. We finally asked whether sTM obtained within the first 5 days of intubation was associated with in-hospital 90-day mortality. Using Cox regression, in both univariate and multivariable models, sTM had a statistically significant association with mortality. For univariate analysis, the HR was 1.003 (95% CI 1.001–1.005, *p* < 0.002) and for multivariable analysis, HR was 1.003 (95% CI 1.000–1.005, *p* = 0.024) for each nanogram/milliliter increase in measured sTM (Table 2). Further, we evaluated an additional model where OI obtained within the first 24 h after intubation was incorporated into a multivariable analyses along with the covariates of age, PRISM-III score, race and sex. sTM was still independently associated with mortality after adjusting for these covariates using Cox proportional hazard regression (HR = 1.003, CI = 1.001–1.006, *p* < 0.01, *n* = 432). We then performed an exploratory analysis of interaction terms to evaluate whether severity of respiratory failure as captured by OI would differentially affect the correlation between sTM and mortality. There was no interaction between OI and sTM for the outcome of mortality by various statistical approaches (*p* = 0.258 by Cox proportional hazard model, *p* = 0.428 by mixed effect modeling, and *p* = 0.358 by logistic regression utilizing day 1 sTM as the predictor variable). Finally, given that 59% of our study population received neuromuscular blockade at day 1, and 50% received vasopressor support at day 1, we evaluated an additional multivariable model incorporating day 1 sTM, Age, Sex, PRISM-III score, vasopressor use and neuromuscular blockade. In this model, day 1 sTM was still independently associated with mortality by logistic regression (OR 1.005, CI 1.001–1.009, *p* = 0.02, *n* = 233, Additional file 1: Table S3).

**Table 2:** Time-dependent multivariate analysis for mortality

Covariates	HR (95% CI)	*P* value
sTM	1.00 (1.00–1.01)	0.024
Age (Years)	1.09 (1.03–1.15)	< 0.01
Sex (Male)	0.85 (0.43–1.66)	0.626
Race (White v not)	0.64 (0.30–1.38)	0.253
PRISM-III Score	1.02 (0.98–1.07)	0.299

Counting process Cox regression analyses were done. sTM between days 0 and 5 were used as the selected predictor variable. Selected covariates as listed. The outcome is death. *n* = 432

### Levels of soluble thrombomodulin correlate with the presence of multi-organ failure

Next, we asked if sTM levels obtained within 5 days of intubation were associated with increased number of non-pulmonary failed organs up to hospital day 28. Out of the 432 patients with sTM collected within 5 days of intubation, 45% (194) experienced non-pulmonary multiorgan failure (2 or more failed organs in addition to the need for ventilation). To evaluate for number of failed organs as an outcome, the rate of change of sTM (slope) and projected sTM at day 0 (intercept) were used as the predictor variables. A multivariable MEM adjusting for age, sex, race (Caucasian vs not) and PRISM-III score revealed that higher starting values of sTM as well as the rate of increase in sTM (i.e., intercept and slope) were associated with an increased number of extrapulmonary failed organs daily up to day 28 (For sTM intercept, Estimate = 0.003, *p* < 0.0001; For sTM slope, Estimate = 0.01, *p* < 0.001, *n* = 386, Table 3).

**Table 3:** Multivariable mixed effect analysis of increase in number of failed organs in the first 28 days

Covariates	Estimate	SE	*P* value
Intercept sTM	2.77 E−3	5.21 E−4	< 1.00 E−4
Slope sTM	1.00 E−2	3.01 E−3	9.00 E−4
Age (years)	2.96 E−2	6.40 E−3	< 1.00 E−4
Sex (male)	4.80 E−2	7.37 E−2	0.51
Race (white)	7.15 E−3	8.50 E−2	0.93
PRISM-III Score	6.03 E−2	5.35 E−3	< 1.00 E−4

Slope of sTM was derived from up to three values of sTM collected within 5 days of enrollment for each patient. Intercept of sTM was derived from the slope. Patients with only one measurement of sTM were excluded. *SE* standard error. *n* = 386

### Soluble thrombomodulin did not correlate with ventilator free days or ICU length of stay

We evaluated if sTM levels correlated with length of stay (LOS) in the pediatric ICU (PICU) or ventilator free days. Competing risk analysis utilizing Cox proportional hazard regression revealed that neither increased slope of sTM nor the sTM intercept (i.e., initial sTM) incurred a statistically significant association with ventilator free days (*p* > 0.4 for slope and intercept, *n* = 430, data not shown). Additionally, Cox proportional hazard analysis revealed no association between sTM and PICU LOS (*p* > 0.4 for sTM slope and intercept, *n* = 430, data not shown).

### Levels of soluble thrombomodulin correlate with worsening oxygenation

We tested the relationship of sTM measured within the first 5 days after intubation with maximum OI values measured or converted from OSI within those 5 days. Only sTM values collected before the peak OI was reached were utilized for this analysis. A unit increase in sTM (1 ng/ml) was associated with a statistically significant increase in OI (Table 4, estimate = 0.015, *p* = 0.01, *n* = 252) after adjusting for age, sex, PRISM-III score and race (caucasian vs. not). Levels of sTM examined on individual days revealed no statistically significant association with maximal OI (data not shown).

**Table 4:** Estimate of effect of sTM on OI/OSI in the first 5 days

Covariates	Estimate	SE	*P* value
sTM	1.51 E−2	5.53 E−3	0.01
Age (years)	−1.77 E−1	1.06 E−1	0.10
Sex (male)	−1.25	1.22	0.31
Race (White v not)	4.53 E−1	1.37	0.74
Prism III Score	1.46 E−1	8.58 E−2	0.09

Multivariable mixed effect model to estimate effect of sTM on OI/OSI in the first 5 days, adjusted for the covariates of age, gender, race and PRISM-III score. sTM was the selected predictor variable. *n* = 252

## Discussion

In this study, higher initial values and rates of increase in soluble thrombomodulin (sTM) were associated with mortality in children with ARF. Moreover, elevated levels of sTM, particularly on day 1, independently associate with increased risk of in-hospital mortality after adjusting for several factors such as age, different markers of disease severity, severity of respiratory failure and use of neuromuscular blockade. There was also a statistically significant association between sTM and worsening oxygenation index, a validated marker of pulmonary dysfunction and ARDS severity [29, 30]. Finally, higher initial values of sTM, and/or a greater rate of increase in sTM, were associated with multi-organ failure.

Thrombomodulin is an attractive candidate for assessment of ARF and ARDS given that the majority of thrombomodulin is found in the lung [22] and its cleaved, soluble form (sTM) can be detected in patient plasma [31]. We observed that at day 1, the area under the ROC curve was 0.7, which suggests moderate usefulness in prognosticating mortality from respiratory failure in this population. In context, this is similar to the AUC for procalcitonin in differentiating between bacterial and viral pneumonia in adults [32]. It will be useful to evaluate the utility of sTM as a prognostic marker in combination with other biologic and clinical markers of ARDS in future studies.

In this study, levels of sTM correlated not only with mortality but also with severity of hypoxic respiratory failure. It is likely that pulmonary vascular damage would be a principal contributor to serum sTM in this study of pediatric acute respiratory failure from primary pulmonary or airways disease. Given the known association of sTM with vascular damage, and the loss of the anti-thrombotic molecule at the site of injury, it is conceivable that elevated sTM may reflect an increase in pulmonary dead space ventilation in ARDS. Dead space is a strong predictor of mortality in ARDS, even surpassing markers that measure oxygenation such as OI and P/F ratio [8, 9, 33]. Since the *RESTORE* trial did not record parameters for dead space ventilation, future studies on sTM would benefit from a prospective evaluation of sTM and dead space ventilation in ARDS or ARF.

Finally, sTM was associated with higher rates of extrapulmonary multiorgan failure. We posit whether this is a consequence of the pro-thrombotic state caused by the cleavage of thrombomodulin. Indeed, recombinant sTM, by replacing the vasculitis-induced depletion of membrane-bound local thrombomodulin, has been implicated in protection or reversal of vascular injury, disseminated intravascular coagulation (DIC) and in animal models of ARDS. In animal studies, recombinant sTM was shown to have a protective effect on septic rats by suppressing leukocyte adhesion to the microvasculature, reducing thrombus formation and preventing endothelial damage [34]; and murine studies have suggested a protective role of sTM in LPS-induced ARDS [35]. In humans, a randomized clinical trial evaluating patients with DIC suggested that treatment with recombinant sTM showed a more significant reversal of DIC than did heparin therapy, but did not evaluate the outcome of mortality [36]. However, a large, multicenter clinical trial testing the therapeutic effect of recombinant sTM on 800 patients with sepsis-associated coagulopathy revealed no effect of sTM therapy on patient mortality, or secondary outcomes such as shock free, dialysis free and ventilator free days [37]. Since the latter study enrolled patients presenting with sepsis complicated by DIC, it is very possible that the population was too heterogeneous to observe an effect on patients that would otherwise benefit from therapy. There was no effort in that trial to enrich for patients with an elevated thrombomodulin plasma level. In contrast, the *BALI* cohort, in which we did find an association of elevated levels of sTM with higher mortality, included only children with a primary respiratory diagnosis. We postulate that since sTM is primarily derived from lung endothelium, patients with respiratory failure may be more likely to show a benefit from recombinant thrombomodulin compared to a population with non-pulmonary sources of sepsis. In addition, given the promising therapeutic effect of recombinant sTM on murine ARDs, it would be important to evaluate the therapeutic role of recombinant thrombomodulin specifically in patients with ARDS demonstrating elevated dead space ventilation and increased sTM as a marker of thrombomodulin depletion from the pulmonary vascular endothelium. Dead space could be measured at the bedside with the ventilatory ratio, an index that is associated with higher mortality in ARDS [10].

The strength of this study lies in its relatively large sample size that includes a diverse study population in children. In addition, the study benefits from the availability of plasma samples from multiple time points and a well curated collection of data elements. The chosen outcomes of mortality, severity of hypoxic respiratory failure and multi-organ failure are of high clinical applicability and are arguably the most useful in assessing patient health. One study limitation is that we did not have access to data on ventilator parameters such as tidal volume and PEEP, which precluded our ability to investigate how ventilator changes may correlate with sTM levels. Another limitation was that all outcomes studied were measured in a population with some subtype of respiratory failure as a primary diagnosis, with almost 70% of the cohort developing PARDS within 5 days of intubation. As such, these findings can only be interpreted in the context of respiratory failure commonly leading to PARDS. Another limitation is that since over 90% of patients who developed PARDS did so by day 1, there was limited opportunity to assess the association of sTM with PARDS development.

## Conclusion

Plasma levels of sTM in pediatric patients receiving ventilatory support were predictive of worsening oxygenation defect, higher mortality and more organ failure. Consequently, sTM may have clinical promise in biomarker guided therapies. Future studies are needed to evaluate whether sTM correlates with worsening dead space ventilation and whether dead space could be reversed in select patients with ARF treated with sTM.

## Supplementary Information

**Additional file 1**. Supplemental methods, tables and legends.

**Additional file 2**. Supplementary Figures.

## Data Availability

The data that support the findings of this study are available from the RESTORE study but restrictions apply to the availability of these data, which were used under license for the current study, and so are not publicly available. Data are, however, available from the authors upon reasonable request and with permission of the RESTORE study.
