# Supplementary material for: Thrombomodulin is associated with increased mortality and organ failure in mechanically ventilated children with acute respiratory failure: biomarker analysis from a multicenter randomized controlled trial
Source: Crit Care. 2021 Aug 3;25:271. doi: 10.1186/s13054-021-03626-1 (PMC8330123; doi:10.1186/s13054-021-03626-1)
Supplement: Supplementary file 1 — Additional file 1. Supplemental methods, tables and legends. [file 13054_2021_3626_MOESM1_ESM.docx]

**Supplemental Methods**

**BALI and RESTORE Trials**

The *BALI* study was designed to collect biological specimens to examine the association of specific plasma protein (including sTM) and genetic biomarkers with PARDS. Guardians for patients were approached for consent for *RESTORE* within 24 h of meeting *RESTORE* study criteria and were approached for consent for participation in *BALI* as soon as possible after consenting for *RESTORE* (often immediately afterwards). Twenty-two of the 31 pediatric intensive care units (PICUs) participating in *RESTORE* also participated in *BALI*. In *RESTORE*, children 2 weeks to 17 years of age treated with invasive mechanical ventilation for acute airways and/or parenchymal lung disease were eligible. The *RESTORE* study excluded children for whom the length of mechanical ventilation was unlikely to be altered by the sedation management protocol (i.e., children who were ventilator dependent on PICU admission or those expected to be extubated within 24 h).(1) There were no additional inclusion or exclusion criteria for the *BALI* study.

**Definition of selected variables and outcomes**

Duration of mechanical ventilation was defined as done in *RESTORE* with patients assigned 28 days if they remained intubated, were transferred or died prior to day 28, therefore making this outcome equivalent to ventilator-free days.

Oxygenation index (OI) was calculated as per the standard definition of mean airway pressure (in cmH_2_O) × FiO_2_ × 100 ÷ PaO_2_. OSI was calculated as mean airway pressure (in cmH_2_O) × FiO_2_ × 100÷SpO_2_. If the OI was not measured for a specific day, it was calculated from the OSI using the following equation OI = (OSI - 2.76) ÷ 0.547(2) as long as the SpO_2_ was ≤ 97%.

sTM values obtained after the maximal OI was attained were excluded, and consequently patients were excluded if there were less than two sTM values collected before the maximal OI was reached.

Organ systems considered in quantifying organ failure were cardiovascular, renal, neurological, hematological and hepatological systems, which was based on a modified Goldstein criteria as previously described.(1) PARDS was defined as described by the Pediatric Acute Lung Injury Consensus Conference(3) except that all patients defined as having PARDS also had bilateral infiltrates within 2 days before or 1 day after meeting OI or OSI criteria for PARDS. The latter modification was necessary because RESTORE did not collect data on patients with unilateral infiltrates.

Supplemental Tables

**Supp. Table 1.** Baseline characteristics of study population.

| **Characteristics** | **N (%)** |
| --- | --- |
| **Sex** |  |
| Female | 198 (45.8) |
| Male | 234 (54.2) |
| **Race** |  |
| White | 313 (72.5) |
| Black/African American | 72 (16.7) |
| Asian | 16 (3.7) |
| Other | 11 (2.5) |
| Multiracial/More than one race | 20 (4.6) |
| **Ethnicity** |  |
| Hispanic or Latino | 102 (23.6) |
| Not Hispanic or Latino | 328 (75.9) |
| Unknown/Unavailable | 2 (0.5) |
| **Primary diagnosis on enrollment** |  |
| Bronchiolitis | 84 (19.4) |
| Asthma or reactive airway disease | 48 (11.1) |
| Pneumonia (any organism) | 159 (36.8) |
| Aspiration pneumonia | 25 (5.8) |
| Thoracic trauma: pulmonary contusion or inhalation burns | 6 (1.4) |
| Acute respiratory failure post BMT | 8 (1.9) |
| Acute respiratory failure related to sepsis | 80 (18.5) |
| Other | 22 (5.1) |
| **PARDS present on day 0** |  |
| Yes | 245 (56.7) |
| No | 187 (43.3) |
| **Age** |  |
| Median (IQR) | 4.1 (0.7-11) |
| **PRISM-III score** |  |
| Median (IQR) | 8 (3.5-13) |

IQR- Interquartile range.

**Supp. Table 2.** Primary cause of death.

| **Primary cause of death** | **N (%)** |
| --- | --- |
| **Respiratory failure** | 17 (3.9) |
| **Multisystem organ failure** | 10 (2.3) |
| **Sepsis/septic shock** | 3 (0.7) |
| **Cancer** | 3 (0.7) |
| **Other** | 2 (0.5) |

**Supp. Table 3.** Day 1 sTM Correlates with Mortality.

| Covariates | OR (95% CI) | P Value |
| --- | --- | --- |
| sTM day 1 | 1.005 (1.001 – 1.009) | 0.02 |
| Age (Years) | 1.115 (1.018 – 1.228) | 0.02 |
| Sex (Male) | 1.300 (0.462 – 3.727) | 0.62 |
| PRISM-III Score | 1.035 (0.961 – 1.111) | 0.35 |
| Vasopressor Use | 1.259 (0.367 – 4.824) | 0.72 |
| Neuromuscular Blockade Use | 3.069 (0.976 – 12.01) | 0.07 |

Multivariable Logistic Regression model using Day 1 sTM as the predictor variable and adjusting for Vasopressor and Neuromuscular Blockade. n=233.

**Supplemental Figure Legends**

Supplemental Figure 1- Venn diagram representing distribution of sampled cohort. Inner circle, in green, represents true study population.

Supplemental Figure 2- sTM values were not significantly different between patients who did or did not have PARDS on individual days. Box plot summary of range of sTM values by day, grouped by PARDS status on that day. (n= 22 and 34 on day 0, 56 and 78 on day 1, 63 and 90 on day 2, and 41 and 77 on day 3 for those without and with PARDS, respectively; p>0.05 for all days as per Mann Whitney U test).

References for supplemental material-

1. Curley MAQ, Wypij D, Watson RS, et al. Protocolized Sedation vs Usual Care in Pediatric Patients Mechanically Ventilated for Acute Respiratory Failure. *JAMA*. 2015;313(4):379. doi:10.1001/jama.2014.18399

2. Whipp BJ. Physiological mechanisms dissociating pulmonary CO 2 and O 2 exchange dynamics during exercise in humans. *Author J Compil C*. 2007;92:347-355. doi:10.1113/expphysiol.2006.034363

3. Jouvet P, Thomas NJ, Willson DF, et al. Pediatric Acute Respiratory Distress Syndrome: Consensus Recommendations from the Pediatric Acute Lung Injury Consensus Conference. In: *Pediatric Critical Care Medicine*. Vol 16. ; 2015:428-439. doi:10.1097/PCC.0000000000000350
