# Supplementary figures and images for: Thrombomodulin is associated with increased mortality and organ failure in mechanically ventilated children with acute respiratory failure: biomarker analysis from a multicenter randomized controlled trial
Source: Crit Care. 2021 Aug 3;25:271. doi: 10.1186/s13054-021-03626-1 (PMC8330123; doi:10.1186/s13054-021-03626-1)

Total Enrolled = 549

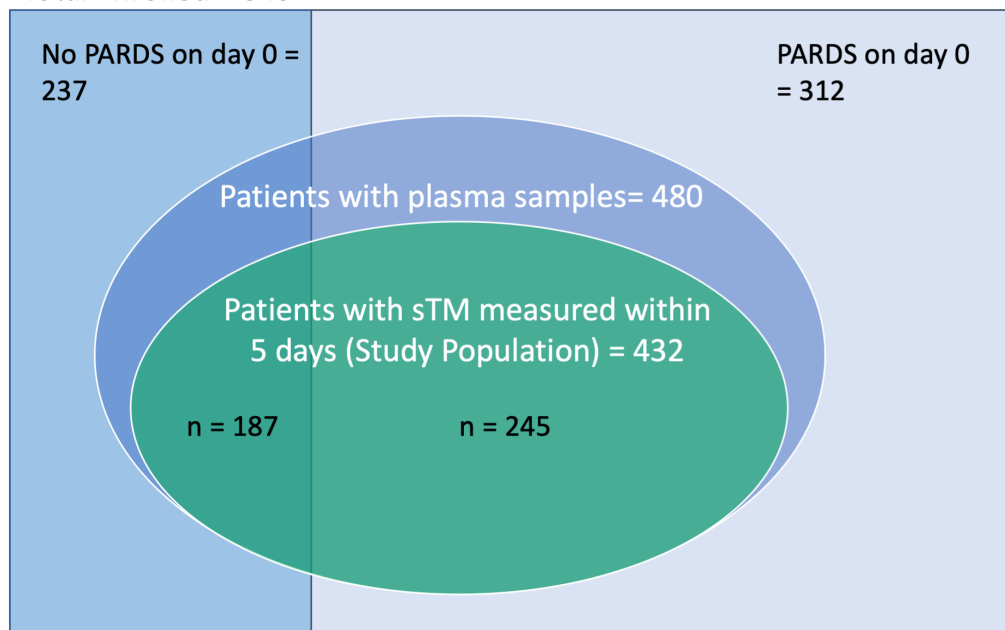

sTM in Patients With and Without ARDS

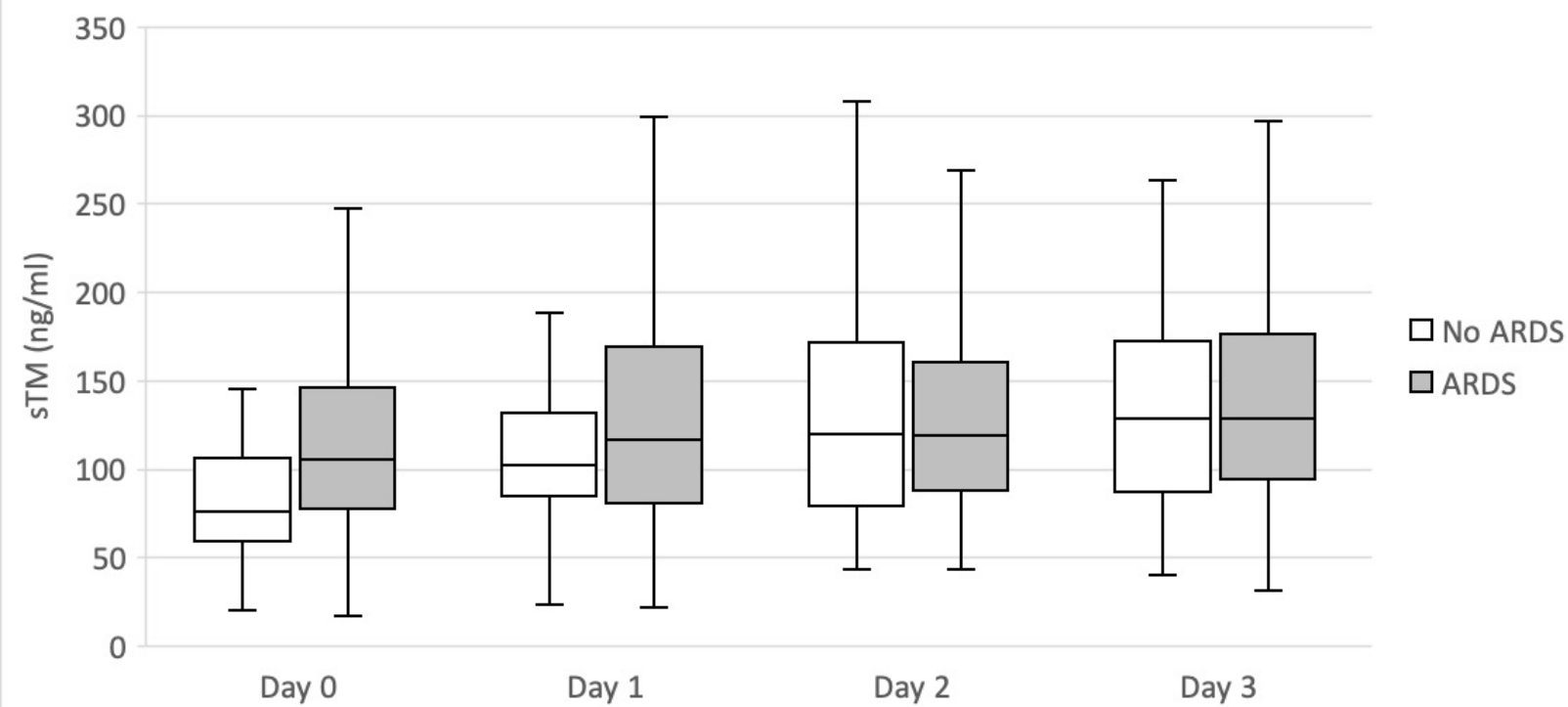

Supplement: Supplementary file 2 — Additional file 2. Supplementary Figures. [file 13054_2021_3626_MOESM2_ESM.pdf]
